# Incidence and risk of cancer emergence among patients post-kidney transplantation in Japan

**DOI:** 10.1007/s10157-025-02748-y

**Published:** 2025-08-27

**Authors:** Saho Kanno, Tatsuya Noda, Tomoya Myojin, Yuichi Nishioka, Shinichiro Kubo, Masahiro Eriguchi, Ken-ichi Samejima, Kazuhiko Tsuruya, Tomoaki Imamura

**Affiliations:** 1 Department of Public Health, Health Management and Policy, Nara Medical University, 840 Shijyo-Cho, Kashihara City, Nara 634-8521 Japan; 2 Department of Community Health and Preventive Medicine, Hamamatsu University School of Medicine, 1-20-1 Handayama, Chuo-Ku, Hamamatsu, Shizuoka 431-3192 Japan; 3 Department of Nephrology, Nara Medical University, 840 Shijo-Cho, Kashihara, Nara 634-8521 Japan

**Keywords:** Kidney transplantation, Post-transplant cancer, Standardized incidence ratio, Administrative claims database-NDB-national database

## Abstract

**Background:**

Cancer is one of the most common complications after kidney transplantation and an important cause of mortality. However, no large, nationally representative study has investigated cancer incidence post-kidney transplantation. This study aimed to determine the standardized incidence ratio (SIR) for cancer after kidney transplantation using the National Database of Health Insurance Claims (NDB).

**Methods:**

We used NDB from April 2013 to March 2022; patients were included if they had been on dialysis for at least one year, were diagnosed with cancer related to post-kidney transplantation, and were prescribed immunosuppressant drugs in FY2014 or FY2015. We defined patients with cancer as those who were coded as ICD-10 for cancer in FY2016 or later. The number of patients and SIRs were tabulated according to the duration after kidney transplantation and cancer type.

**Results:**

The total number of patients was 4484 (males: 2879; females: 1605). The SIRs of all cancers from the first to the seventh year after kidney transplantation were 232/291/235/248/257/187/149, respectively, showing a gradual downward trend over time. The predilection sites of cancer in both men and women were post-transplant lymphoproliferative disease, Kaposi sarcoma, and the kidney.

**Conclusion:**

This observational study, which followed over 100 million people, is the first large-scale research to track kidney transplant recipients for under 10 years. It incorporates an unprecedented sample size and uniquely identified short-term cancer risk trends following kidney transplantation.

**Supplementary Information:**

The online version contains supplementary material available at 10.1007/s10157-025-02748-y.

## Introduction

Kidney transplantation is one of the kidney replacement therapies for patients with end-stage kidney disease, along with hemodialysis and peritoneal dialysis. Kidney transplantation is considered an ideal treatment, with fewer time constraints, higher quality of life, and lower mortality compared to hemodialysis. Recent advances in immunosuppressants have improved organ transplantation outcomes. According to a 2023 report by the Japanese Society for Clinical Renal Transplantation (JSCRT) and the Japan Society for Transplant (JST), the 10 year graft survival rate (living donor kidney) was 90.6% in 2010, and the live organ transplantation rate was 81.3%, both remarkable improvements in performance since 2001 [1].

Cancer and viral infections have been reported as complications of kidney transplantation. In Japan, cardiac diseases, infectious diseases, and malignant tumors are among the three leading causes of death among recipients [1]. In recent years, since 2010, cardiac disease has been decreasing probably due to improved recipient management, and cancer has been found to be the leading cause of death [1].

Recent reports from a single center in Japan indicate that the relative risk of cancer is six times higher in patients with kidney transplantation than in the general population. Therefore, as the number of aging patients with kidney transplantation increases, the incidence of malignant tumors is expected to increase [2].

In previous studies, the standardized incidence ratio (SIR) before and after kidney transplantation was 3.27 (mean follow-up 8.5 years), 2.5 (cumulative for all periods), 3.9 (cumulative for all periods), and 1.5 (cumulative for all periods) for all cancers except skin cancer [3–6]. The study also reported increases in post-transplant lymphoproliferative disease (PTLD), skin, breast, kidney, colon, stomach, uterus, prostate, liver, tongue, urinary tract, thyroid, pancreas, lung, ovary, anus, and vagina [7, 8]. The predilection sites of cancers in the U.S. are skin, lymphoma, kidney, lip, and more recently, particularly in carcinomas involving viruses, while in Japan, kidney, liver, stomach, breast, colon, and malignant lymphoma are reportedly more common [9].

Internationally, most studies examining the risk of cancer incidence after kidney transplantation have been limited by small sample sizes, short-term follow-ups, and specific sites; only a few recent studies have examined the risk on a national scale. With the increasing number of kidney transplantation and survival rates, a highly accurate picture of cancer incidence after transplantation is expected to be an important decision-making criterion for patients with end-stage kidney disease and for clinicians involved in kidney transplantation.

Therefore, we focused on specific cancer types (PTLD, lymphoma, skin, Kaposi's sarcoma, lip, thyroid, tongue, lung, stomach, liver, pancreas, colon, anus, kidney, urinary tract, prostate, breast, uterus, ovary, vagina, and soft tissue) observed after kidney transplantation in Japan and Western countries and used the NDB to identify cancer incidence risk among kidney transplant recipients. This study aimed to determine the risk of cancer in kidney transplant recipients using data from the NDB and is the first study to use nationally representative large-scale data.

## Methods

### Data

Medical (inpatient), medical (non-inpatient), DPC, and dispensing claims from the NDB were analyzed from April 2013 to March 2022. The NDB is one of the largest and most comprehensive databases in the world, containing information on insured medical care, specified health checkups, etc., for almost all of the population, regardless of the type of health insurance [10].

### Patient definitions

Our previous studies showed that it is difficult to identify kidney transplantation practices for patients with NDB. To reliably identify patients post-kidney transplantation, we defined them as those who were prescribed immunosuppressants, which are always prescribed after kidney transplantation [11].

**Fig. 1:**
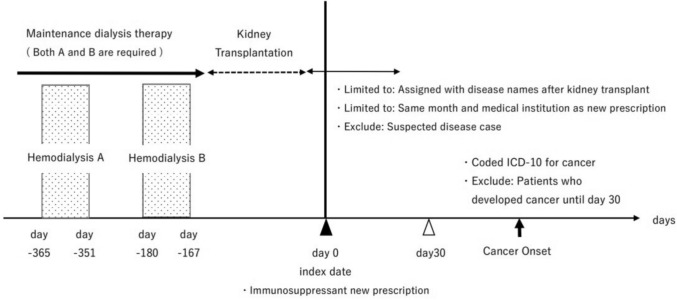
Design diagram: Patients identification and follow-up

The timeline for identifying cancer patients with kidney transplantation after maintenance dialysis therapy is shown in Fig. 1.

#### Aggregation of patients with kidney transplantation after dialysis

To standardize patient backgrounds, those who underwent kidney transplantation after maintenance dialysis therapy for at least one year were included and defined as “patients with kidney transplantation after maintenance dialysis therapy” in this study. Since hemodialysis is administered three times a week, the index date (Day 0) was set as the time when"post-kidney transplantation"(excluding suspected disease) was coded and"immunosuppressant"was prescribed in the same claim. Additionally, the time period between Day-365 to Day-351 (two weeks before one year) and Day-180 to Day-167 (two weeks six months prior patients with at least three dialysis sessions were identified as those whose dialysis had continued for at least one year [12]. The codes for dialysis are in Supplementary Table S1. Disease codes related to kidney transplantation and drug codes for immunosuppressant are in Supplementary Tables S2 and S3, respectively. The number of patients who met the above criteria at least once was counted according to year, age, and sex. The age groups were defined in 10 year increments. In addition, to examine the validity of the data from NDB, we compared with the data from JSCRT and JST. Patients who resumed dialysis after graft loss, and those who required dialysis due to delayed graft function, were also included in the study cohort.

#### Aggregation of cancer patients with kidney transplantation after maintenance dialysis therapy

Patients who met the definition of"kidney transplantation after maintenance dialysis therapy"in FY2014 and FY2015 were followed for up to six years, of which the patients who were coded ICD-10 for cancer in FY2016 or later were counted; the codes are listed in Supplementary Table S4. The following cancer sites, reported to have occurred after kidney transplantation in previous studies, were included in this study: PTLD, lymphoma, skin, Kaposi's sarcoma, lip, thyroid, tongue, lung, stomach, liver, pancreas, colon, anus, kidney, urinary tract, prostate, breast, uterus, ovary, vagina, and soft tissue; the codes are listed in Supplementary Table S4.

Patients with undiagnosed cancer at the time of kidney transplantation and those who developed cancer within 30 days after kidney transplantation were excluded [4, 5]. Patients who were coded an ICD-10 for the first time during the"less than 1 year","1–2 years","2–3 years","3–4 years","4–5 years","5–6 years","6–7 years", and"total for all periods"kidney transplantation after maintenance dialysis therapy were included.

### SIR for cancer patients with kidney transplantation after maintenance dialysis therapy

SIRs were calculated for each cancer site and postoperative period to compare the cancer incidence rates between kidney transplant and non-transplant patients. The age criterion was April 1, 2014. The expected number of patients with cancer was calculated from the cancer incidence rate among patients coded as ICD-10 for cancer. The number of patients with each cancer type was divided by the expected number of patients with cancer, and sex- and age-adjusted SIRs were calculated.

$$\text{SIR}=\frac{The number of cancer pstients after \text{kidney transplant and dialysis}}{Sumof\left[\left(cancerincidenceintheexaminedpatientpopulation\right)\times \left(numberofcancerpstientsafter\text{kidneytransplantanddialysis}\right)\right]by age by year}$$

## Results

### Number of patients with kidney transplantation after maintenance dialysis therapy

Table 1 shows the difference between the number of patients with kidney transplantation after maintenance dialysis therapy by sex and year and the number reported by the JSCRT and JST. The total number of patients in the previous year from FY2014 to FY2021 was 4484 for men and women, 2879 for men and 1605 for women. The difference between the numbers reported by the JSCRT and JST ranged from − 14.19 to 3.88%, and the total was − 5.74%, indicating that the NDB total was less than the number reported by the JSCRT and JST in many years. Over time, the number of patients decreased once in FY2020 for both men and women and has been slowly increasing since then.

**Table 1:** The difference between the number of patients with kidney transplantation after maintenance dialysis therapy by sex and year and the number reported by the JSCRT and JST

	The number of patients								
Year	2014	2015	2016	2017	2018	2019	2020	2021	Total
NDB (Men)	379	329	394	368	367	404	317	321	2879
NDB (Women)	226	196	210	195	219	227	148	184	1605
NDB (Total)	605	525	604	563	586	631	465	505	4484
JSCRT and JST	606	585	581	626	616	661	536	538	4749
The percentage (%)	− 0.17	− 10.81	3.88	− 10.60	− 4.99	− 4.64	− 14.19	− 6.33	− 5.74

‘‘The percentage (%)” describes the difference between NDB and JSCT, JST

*NDB* The National Database of Health Insurance Claims and Specific Health Checkups of Japan, *JRDR* Japanese Society for Dialysis Therapy renal data registry, *JST* The Japan Society for Transplant

**Fig. 2:**
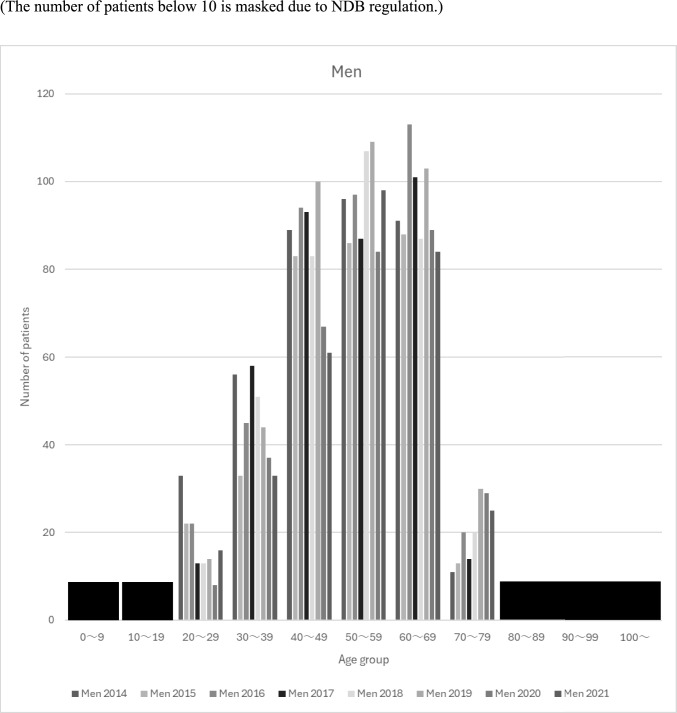
The numbers of patients with kidney transplantation after dialysis according to sex and age groups (The number of patients below 10 is masked due to NDB regulation)

**Fig. 3:**
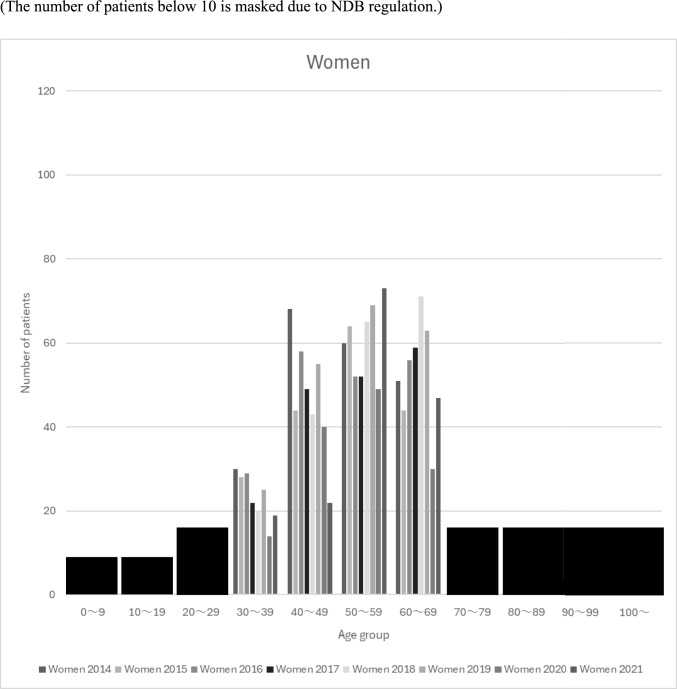
The numbers of patients with kidney transplantation after dialysis according to sex and age groups (The number of patients below 10 is masked due to NDB regulation)

The numbers of patients with kidney transplantation after maintenance dialysis therapy according to sex and age groups are shown in Figs. 2 and 3, respectively. Patient numbers peaked in the 40–60 age group for both sexes, with all age groups showing a decline in 2020. The patient population showed divergent gender trends: while the number of male patients declined across most age groups except those in their 50 s, the number of female patients in their 50 s experienced only a temporary decrease in 2020 before returning to an upward trajectory.

### Standardized incidence ratio (SIR)

The SIRs by years since kidney transplantation are shown in Table 2. The SIRs for all years were 232 for both men (n = 228) and women (n = 239). By year, there was a gender difference, with higher SIR for males in the"1–2 years"group and for females in the"less than 1 year"group. The number of cases decreased gradually over time in both men and women. Table 3 shows the SIR according to the cancer site. The predilection of cancer sites for both men and women were PTLD, Kaposi's sarcoma, and kidney, and the SIRs for PTLD and Kaposi's sarcoma were extremely high compared to those for other sites.

**Table 2:** The SIRs by years since kidney transplantation

Period	Total			Men			Women		
	SIR	SIR95%CI		SIR	SIR95%CI		SIR	SIR95%CI	
		Low	High		Low	High		Low	High
1 <	232	147	349	186	96	325	320	159	572
1 ~ 2	291	192	424	336	205	519	210	85	434
2 ~ 3	235	144	364	221	114	385	262	113	516
3 ~ 4	248	151	382	252	134	431	240	97	495
4 ~ 5	257	157	397	285	156	477	210	77	457
5 ~ 6	187	102	314	170	73	335	216	79	469
6 ~ 7	149	71	274	120	39	280	197	64	459
Total	232	195	275	228	182	283	239	177	315

*SIR* standardized incidence ratio

**Table 3:** The SIRs according to the cancer site

Rank	Total				Men				Women			
	Site	SIR	SIR95%CI		Site	SIR	SIR95%CI		Site	SIR	SIR95%CI	
			Low	High			Low	High			Low	High
1	PTLD	81,419	16,791	237,942	PTLD	80,337	9729	290,205	KS	494,538	12,521	2755,391
2	KS	58,120	7039	209,949	KS	30,874	782	172,020	PTLD	83,674	2118	466,199
3	Kidney	1090	623	1770	Kidney	1006	520	1757	Kidney	1456	397	3728
4	Skin	580	265	1102	Skin	690	278	1423	Urinary tract	1451	532	3158
5	Urinary tract	567	317	935	Thyroid	552	114	1614	LYM	575	263	1092
6	PTLD and LYM	465	291	704	PTLD and LYM	410	219	702	PTLD and LYM	575	263	1091
7	LYM	423	258	653	Urinary tract	403	184	765	Pancreas	399	82	1167
8	Uterus	259	104	533	LYM	348	173	622	Skin	372	45	1345
9	Liver	230	75	537	Soft tissue	298	8	1662	Lung	260	71	665
10	Lung	213	117	358	Liver	229	62	587	Uterus	259	104	535
11	Thyroid	200	41	584	Lung	199	95	366	Liver	234	6	1306
12	Prostate	190	95	340	Prostate	190	95	340	Stomach	196	40	572
13	Soft tissue	186	5	1,035	Stomach	103	38	225	Breast	147	67	279
14	Pancreas	158	43	406	Colon	72	26	156	Colon	96	20	280
15	Breast	146	67	276	Pancreas	56	1	314	Lip	0	0	0
16	Stomach	123	56	233	Lip	0	0	0	Thyroid	0	0	0
17	Colon	78	36	148	Tongue	0	0	0	Tongue	0	0	0
18	Lip	0	0	0	Anus	0	0	0	Soft tissue	0	0	0
19	Tongue	0	0	0	–	–	–	–	Ovary	0	0	0
20	Ovary	0	0	0	–	–	–	–	Anus	0	0	0
21	Anus	0	0	0	–	–	–	–	Vagina	0	0	0
22	Vagina	0	0	0	–	–	–	–	–	–	–	–

*SIR* Standardized incidence ratio, *PTLD* Post-transplant lymphoproliferative disease, *KS* Kaposi sarcoma, *LYM* Lymphoma

## Discussion

In this study, using one of the world's largest claims databases, we succeeded in determining the number of patients with kidney transplantation after maintenance dialysis therapy and the risk of cancer after kidney transplantation in Japan in recent years. In addition, no previous study in recent years has followed up kidney transplant recipients for less than 10 years and observed the status of cancer incidence at one-year intervals, thus revealing, for the first time, a trend in the risk of cancer incidence in the short term after transplantation.

### Characteristics of patients with kidney transplantation after maintenance dialysis therapy

Looking at the number of patients with kidney transplantation after maintenance dialysis therapy over time, the number of both male and female patients showed an increasing trend prior to 2020, but decreased after 2020, and then began to increase slowly. This trend is consistent with reports by the JSCRT and JST, and the increase before 2020 is thought to be due to an increase in the number of unrelated transplants, blood type-incompatible transplants, and transplantation in the elderly [13]. However, the decrease in 2020 is speculated to be mainly due to a decrease in the number of surgeries and an increase in the deaths of dialysis patients due to the outbreak of new coronavirus infection (COVID-19) [14]. In addition, several recent reports on the usefulness of preemptive kidney transplantation (PEKT) suggested that an increase in the number of non-dialyzed patients may influence a gradual increase in the number of patients after 2020.

The number of patients by age group showed a broad distribution between the 40–60 age group, with a significant increase in 2021, especially in the 50 s. This trend is consistent with reports from the JSCRT and JST. In contrast, the NDB data for maintenance hemodialysis patients in 2021 showed an increase in all age groups except the 40 s, whereas the reports by JSCRT and JST showed an increase in all age groups except the 60 s. This suggests a decrease in patients with kidney transplantation after maintenance dialysis therapy in the 40 s and an increase in the 60 s. Previous studies examining trends in the number of dialysis patients have reported a decrease in younger dialysis patients and an increase in older dialysis patients [14], and the NDB may reflect these changes in the age distribution of dialysis patients to some extent.

Analysis by age group and sex also showed a trend toward more males than females, which is consistent with reports by the JSCRT and JST. The reason for the higher number of patients with kidney transplantation after maintenance dialysis therapy in men is unclear; however, a report from the Japanese Society for Dialysis Therapy (JSDT) suggested that men have more end-stage kidney disease and dialysis patients, which may lead to a higher number of patients with kidney transplantation after maintenance dialysis therapy [15, 16]. In addition, a Norwegian study reported that the rate of kidney function decline in men increases with age. In contrast, male patients showed a decreasing trend in most age groups, except for those in their 50 s, while female patients showed an increasing trend in most age groups, except for those their 40 s. Although the exact reason for the recent decline in male patients is unclear, Nakai et al. reported that it may be related to the fact that the COVID-19 epidemic temporarily discouraged patients with CKD from visiting medical institutions before the introduction of dialysis in 2020 [14].

### Risk and characteristics of cancer patients with kidney transplantation after maintenance dialysis therapy

Although previous studies on the risk of cancer with kidney transplantation after maintenance dialysis therapy have been conducted both domestically and internationally, there are no studies based on recent nationwide data from Japan. In addition, previous studies have focused on follow-up periods of 5–10 years after transplantation, and none have been conducted in Japan in one-year increments immediately after transplantation. This study is significant in that it is the first to identify trends in cancer incidence within a short period after transplantation.

In this study, the SIRs for most cancer sites were high, confirming a high risk of cancer with kidney transplantation after maintenance dialysis therapy. The SIRs for all cancers over the entire time period were similar: 228 for men and 239 for women; however, by time period, the SIRs were highest in the first two years after transplantation for men and less than one year after transplantation for women. This suggests that there are differences between men and women during the period with the highest risk of cancer incidence. Thereafter, the SIRs remained unchanged in both sexes until approximately 5 years after transplantation, after which they showed a decreasing trend. Although no studies in Japan have examined the SIR at 1-year intervals, Claire et al. and Villeneuve et al. also reported that the SIR was highest at less than 1 year, followed by a leveling off or decreasing trend. Despite racial differences, a consistent trend toward peak risk in the year immediately after transplantation has been observed [3, 4]. Possible factors contributing to this sex difference in SIRs include age at transplantation, the peak incidence age for each cancer type, and sex-related differences in cancer-prone organs.

As sites of cancer affected, PTLD, Kaposi's sarcoma, kidney cancer, and skin cancer showed high SIRs in both sexes, and women showed a particularly high SIR for uterine cancer. Skin cancer, lymphoma, kidney cancer, and lip cancer have been reported as predilection sites in Western studies. However, the risk of virus-related cancers (such as cervical and vaginal cancers caused by HPV) has increased in recent years [9]. Virus-related cancers include the association between EBV and non-Hodgkin's lymphoma, HHV-8 and Kaposi's sarcoma, HPV, and cervical and vaginal cancers; a similar trend was shown in our study for uterine cancer [17, 18].

Although few previous studies have investigated the predilection sites for cancer in Japan, thus limiting comparisons, there were some that were consistent and others that were not in organ-specific SIRs. For example, while the risk of skin and kidney cancers was identified, it was not as high as that in Europe and the U.S., and gastric and other digestive cancers are considered more common; this study found very high SIRs for skin and kidney cancers [19]. Furthermore, for Kaposi's sarcoma, which is believed to be influenced by regional and racial factors, our study showed a high SIR, confirming a trend consistent with that of previous domestic and foreign studies. However, for some cancers, the trend was similar to that observed in Europe and the U.S., suggesting that recent changes in the living environment may influence the incidence of these cancers.

Although a type-specific analysis of immunosuppressants was not performed in this study, previous studies suggest that they affect preferred cancer sites. The use of cyclosporine has increased in recent years and is associated with higher incidence of lymphoma, Kaposi's sarcoma, and kidney cancer. Post-transplant guidelines recommend tacrolimus and cyclosporine as first-line agents [20], which may raise the risk of certain cancers [21]. Further analysis of immunosuppressants in this study could clarify background factors influencing cancer risk.

## Limitations

This study has several limitations based on the NDB tabulations, and the difference between the NDB tabulations and JSCRT/JST reported values ranged from − 14.19% to 3.88%, totaling − 5.74%, indicating that NDB values were lower. One factor contributing to this underestimation is that the NDB does not include data on medical assistance recipients such as welfare recipients who represent 1.6% of the population. Additionally, while academic society reports are based on calendar years, the NDB uses fiscal years, potentially causing discrepancies. Conference reports may also include duplication; for example, the JSDT registers dialysis patients per hospital, and patients moving across prefectures may be counted multiple times [22].

Second, regarding the definition of cancer, the number of patients in this study may have been overestimated because only disease names (ICD-10-coded) were used in the tabulations. Studies using claim data suggest that patient definitions should be performed with caution, and the diagnostic validity of patients with cancer needs to be verified [23, 24]. However, previous studies have shown that the validity of cancer diagnoses is higher in claim data than that of other diseases [25]. Despite these findings, the cancer patient classification system requires further refinement to account for cancer-specific medications, prescription durations, and expert clinical input.

An additional factor contributing to overestimation was cancer observed immediately after transplantation. This is most likely due to the increased screening frequency after transplantation [4]. Therefore, cancers developing within 30 days post-transplant were excluded [5]. Regarding the possibility of cancer already present before transplantation, the recipient indication criteria of the JST clearly state"no malignancy", but it is difficult to definitively determine whether 30 days is appropriate as a criterion for excluding cancer. Also, some patients may have undergone transplantation without a confirmed cancer diagnosis or in exceptional cases. The current dataset lacks clinical details to evaluate these possibilities, making further investigation unfeasible within this study.

Third, regarding the observation period, this study is limited to a 6 year follow-up period. It has been reported that, on average, the development of cancer after kidney transplantation occurs more than 10 years after transplantation. Extended follow-up periods of 10–20 years would likely reveal clearer patterns in both the SIR and preferred sites of cancer development following kidney transplantation.

Finally, although total dialysis duration before transplantation was not considered. As it may affect cancer risk and prognosis, future studies should control for it when assessing the effects of transplantation and immunosuppression.

Due to inherent limitations of the NDB, including the lack of patient background and clinical data that may act as confounders, more refined analyses were not feasible. These limitations should be considered when interpreting the study findings.

## Conclusion

This observational study, which followed over 100 million people, is the first large-scale research to track patients with kidney transplantation after maintenance dialysis therapy for under 10 years. It boasts an unprecedented sample size and uniquely identified short-term cancer risk trends with kidney transplantation after maintenance dialysis therapy.

## Supplementary Information

Below is the link to the electronic supplementary material.

Supplementary file1 (XLSX 26 KB)
